# Human spiruridiasis due to *Physaloptera* spp. (Nematoda: Physalopteridae) in a grave of the Shahr-e Sukhteh archeological site of the Bronze Age (2800–2500 BC) in Iran

**DOI:** 10.1051/parasite/2017019

**Published:** 2017-06-02

**Authors:** Mahsasadat Makki, Jean Dupouy-Camet, Seyed Mansour Seyed Sajjadi, František Moravec, Saied Reza Naddaf, Iraj Mobedi, Hossein Malekafzali, Mostafa Rezaeian, Mehdi Mohebali, Faranak Kargar, Gholamreza Mowlavi

**Affiliations:** 1 School of Public Health, Tehran University of Medical Sciences District 6, Pour Sina St P.O. Box 6446 Tehran 14155 Islamic Republic of Iran; 2 ACMSFI, Hôpital Cochin 27 Faubourg St Jacques 75014 Paris France; 3 Cultural Heritage and Tourism Research Institute, Opposite Iran National Museum 30 Tir St., Imam Khomeini (RA) avenue Tehran Islamic Republic of Iran; 4 Institute of Parasitology, Biology Centre of the Czech Academy of Sciences Branišovská 31 České Budějovice 370 05 Czech Republic; 5 Department of Parasitology, Pasteur Institute of Iran (IPI) No. 69, 12th Farwardin ave 1316943551 Tehran Islamic Republic of Iran; 6 Shaheed Rajaei Cardiovascular Medical and Research Center Valiasr ave Niayesh Intersection Tehran Iran

**Keywords:** Paleoparasitology, Human spiruridiasis, Shahr-e Sukhteh, Iran, *Physaloptera* spp.

## Abstract

Evidence of rare human helminthiasis in paleoparasitological records is scarce. we report here the finding of *Physaloptera* spp. eggs in a soil sample collected in the pelvic and sacrum bones area of a skeleton excavated from a grave of Shahr-e Sukhteh archeological site dating back to the Bronze Age. The site is located in southeastern Iran and has attracted the attention of numerous archeological teams owing to its vast expanse and diverse archeological findings since 1997. The spirurid nematodes *Physaloptera* spp. are rarely the cause of human helminthiasis nowadays, but this infection might not have been so rare in ancient populations such as those in the Shahr-e Sukhteh. Out of 320 skeletons analyzed in this study, only one parasitized individual was detected. This surprising result led us to suspect the role of nematophagous fungi and other taphonomic processes in possible false-negative results. This is the first paleoparasitological study on human remains in this archeological site and the first record of ancient human physalopterosis in the Middle East.

## Abstract (fr)

Les preuves d’infestation humaine par des espèces rares d’helminthes sont rares en paléoparasitologie. Nous rapportons ici l’identification d’œufs de *Physaloptera* spp dans des échantillons de sol prélevés dans la région pelvienne et le sacrum d’un squelette d’une tombe du site archéologique de Shahr-e Sukhteh datant de l’âge du Bronze. Ce site est situé dans le sud-est de l’Iran et a attiré l’attention de nombreuses équipes archéologiques depuis 1997, en raison de son extension et de l’importance des pièces archéologiques découvertes. Les nématodes spiruridés *Physaloptera* spp. sont la cause d’helminthiases humaines rares aujourd’hui mais ceci n’était peut-être pas aussi rare dans les populations anciennes telles que celle de Shahr-e Sukhteh. Sur 320 squelettes analysés dans cette étude, un seul individu a été retrouvé parasité. Ce résultat surprenant nous conduit à soupçonner le rôle des champignons nématophages et d’autres processus taphonomiques dans la survenue d’éventuels résultats faussement négatifs. Ceci est la première étude paléoparasitologique sur des restes humains dans ce site archéologique et le premier cas paléoparasitologique de spiruridose humaine au Moyen-Orient.

## Introduction

Studying biological remains such as coprolites, burial soils, and latrine sediments obtained from archeological sites provides valuable information concerning paleo diet and/or unusual food items associated with parasitic infections in ancient times [1]. Recognizing parasite life cycles *vis-à-vis* their environment, host specificity, and personal behavior sheds light on arthropods as intermediate hosts and their role in the transmission of specific parasites to humans.

A review of the literature on paleoparasitology illustrates the occurrence of certain parasitic infections among our ancestors in communities in the distant past [3, 13]. The Shahr-e Sukhteh (“Burnt City” in Persian) archeological site (30° 39′ N; 61° 24′ E) in southeastern Iran was discovered in the early 20th century, and the initial portion of the excavation project was performed by Maurizio Tosi, an Italian archeologist, in 1967 [27]. This archeological site is representative of the Bronze Age (3200–1800 BC) in the southeastern plateau of Iran and the items excavated from this site support the presence of societies with developed agriculture and animal husbandry as well as metallurgy at that time [15]. The site territory covers about 151 ha and comprises three distinct areas of residential, industrial and cemetery sections. We had the opportunity to analyze human burial soil from this location, and we report here the finding of *Physaloptera* eggs, an infrequent human parasite but a common parasite of dogs and cats.

## Materials and Methods

### Archeological site

**Figure 1.**
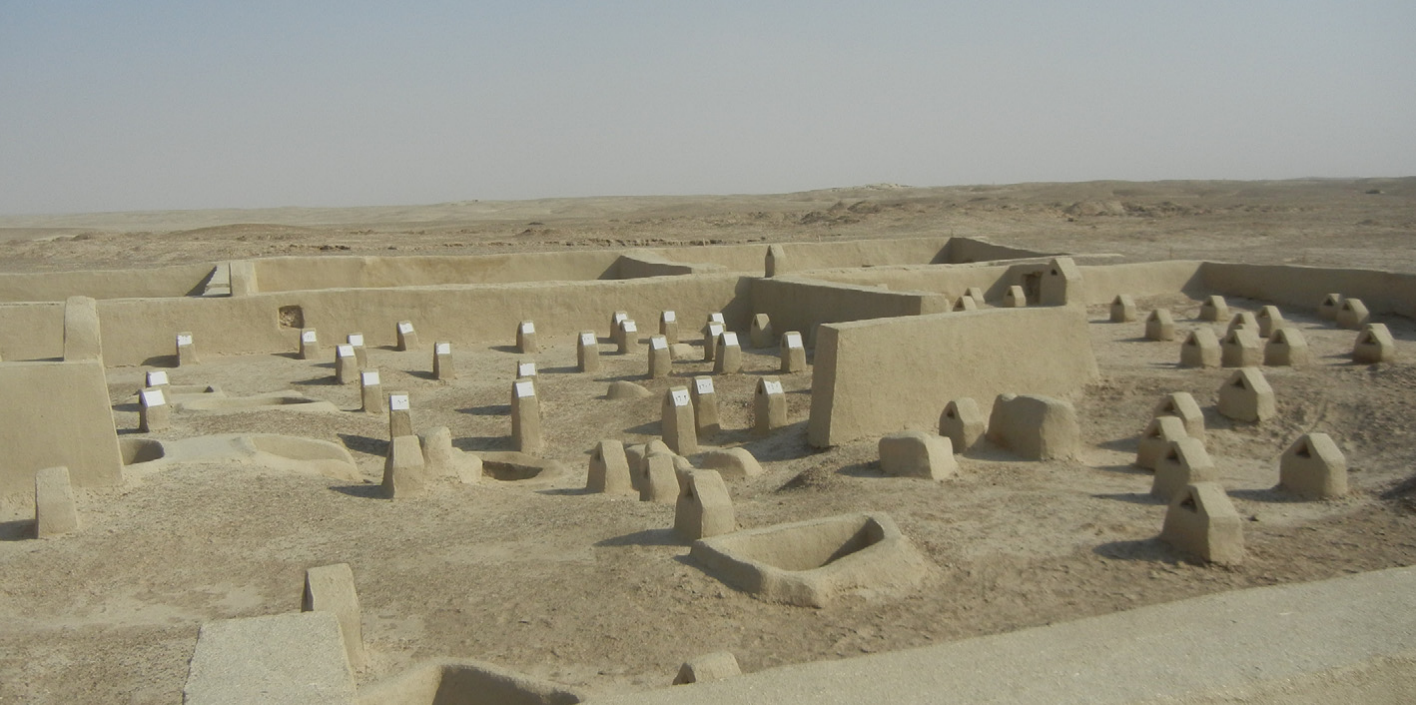
A part of the necropolis in Shahr-e Sukhteh archeological site.

The cemetery section of Shahr-e Sukhteh covers about 25 ha (Fig. 1) and includes around 25,000–40,000 graves [23]. Stored pelvic and sacrum bones, from 320 graves, unearthed in previous excavations since 1997 were used in this study. The age of the skeletons was calculated using the ^14^C method on charcoal obtained from the relevant layers along with the cultural context attribution [24].

### Sampling and microscopic analysis

**Figure 2.**
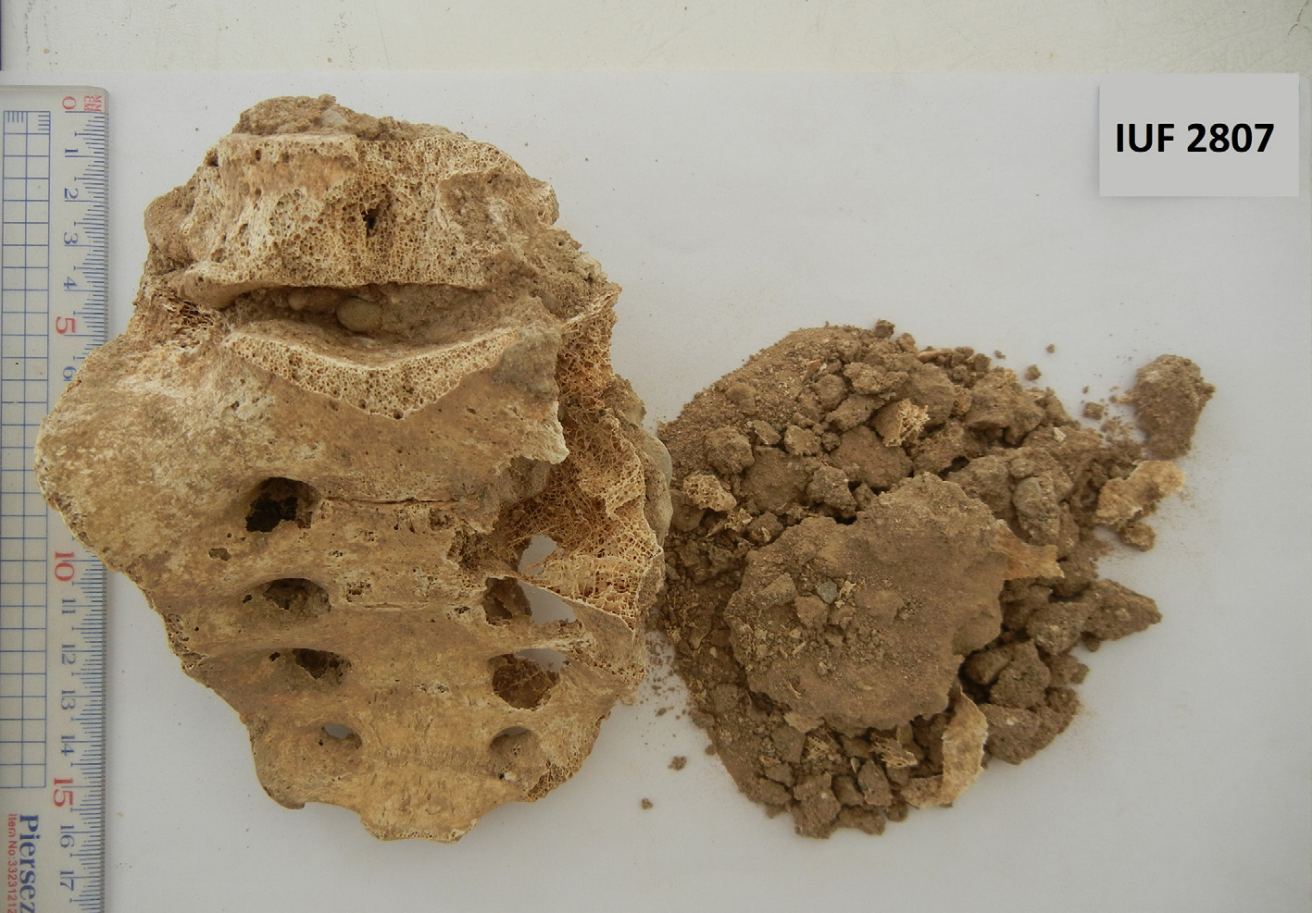
A sacrum showing foramina from which soil samples were examined (burial IUF 2807).

The soil attached to the surface of the bones, specifically the sacrum foramina (Fig. 2), was collected meticulously and kept in plastic zip bags with the same skeleton code. Two 1 g samples from the collected soils were rehydrated in a trisodium phosphate (TSP) solution [22]. Ten days later, microscopic slides were prepared and mounted in glycerin jelly using double 22 × 22 mm cover slides to check for the presence of helminth eggs as reliably as possible. The retrieved helminth eggs were detected by 100×, 400×, and 1000× magnification, respectively, and photographed using a camera-equipped microscope (LABOMED LX 500). The eggs were identified based on morphologic features and morphometric parameters available in reliable references [2, 21, 28].

## Results

**Figure 3.**
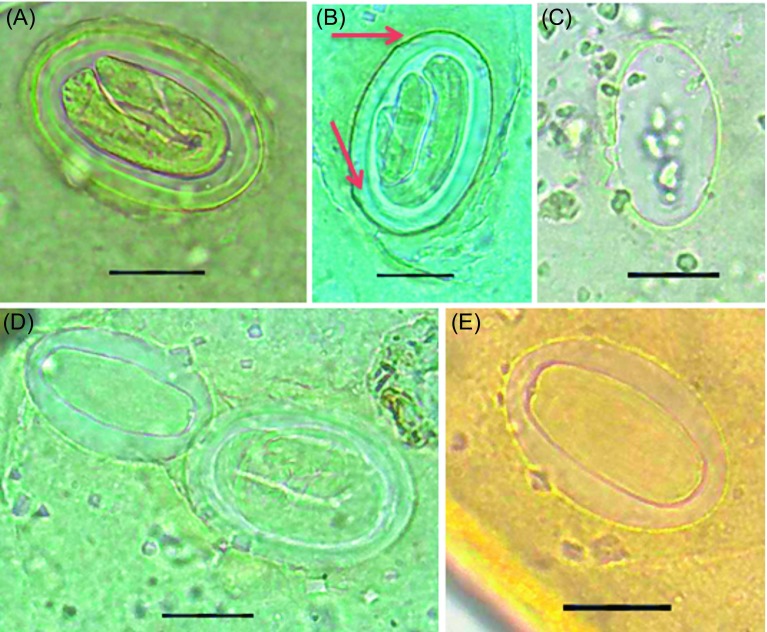
The six *Physaloptera* spp. eggs found in the soil sample. Note the embryos in A, B, D. Arrows in (B) show the considerable thickness of the egg shell. The diagnosis cannot be made with certainty for the egg in (C). Scale bars = 20 μm. Egg shows a hyalinized state of its content (E).

In total, 6711 microscopic slides were thoroughly examined. Out of 320 skeletons analyzed in this study, eggs were detected in soil samples collected from bones of only one skeleton. In one sample (burial IUF 2807, a middle-aged male adult of 35–40 years), six eggs measuring a mean length of 47.9 ± 5.7 and a mean width of 31.5 ± 5.2 μm were collected and were subsequently identified as eggs of *Physaloptera* spp., a very rare group of spirurid worms in humans. The sizes and the morphological features of *Physaloptera* spp. eggs exhibiting smooth, thick shells and double counter in addition to the embryo inside the eggs were all in favor of the present diagnosis. The sizes were also compatible with those described in the literature (Table 1, Fig. 3).

**Table 1.** Measurements of the identified eggs.

Egg	No	Length (μm)	Width (μm)
	1	55.4	35.4
	2	53.8	38.5
*Physaloptera* spp.	3	45.7	30.6
	4	50.4	34.8
	5	42.9	27.2
	6	39.6	23
	Mean ± *SD*	47.9 ± 5.7	31.5 ± 5.2

## Discussion

The genus *Physaloptera* Rudolphi, 1819 is composed of several species parasitizing a broad range of hosts, including mammals, birds, reptiles, amphibians, and insects. Most species utilize insects such as crickets, cockroaches, and beetles as intermediate hosts [6]. *Physaloptera* spp. are also known to infect baboons and chimpanzees as well as other simian primates and the genus was first identified in humans from the Caucasus Mountains in Russia in 1902 [14, 17]. It has also been identified in humans in Africa and South America [26]. Humans acquire the infection by the accidental ingestion of infected arthropods. *Physaloptera* spp. adults are anchored in the esophagus, stomach, and small intestine and can provoke nausea and diarrhea. Sometimes adults are expelled by vomiting and can be confounded with *Ascaris,* though *Phylasoptera* (up to 100 mm in maximum range) are smaller in size [2]. At first glance, the present eggs drew our attention to the eggs of more common parasites such as *Ascaris lumbricoides* and/or *Capillaria* spp.

It appears that decorticated and immature eggs of *A. lumbricoides* can be confused in shape with spirurid eggs [6, 17]. In the present study, the deep concentration in the morphology of the eggs in terms of their oval shape and thick-walled appearance together with the larvae inside in three of the eggs (Figs. 3A, 3B and 3D) and the visible “double counter” in one egg (Fig. 3B) were in favor of *Physaloptera* spp. eggs. The parameters were compatible with reference measurements for *Physaloptera* eggs: 44–65 by 32–45 for Beaver et al. [2]; 46–51 by 33–37 for Vandepite et al. [28]. However, the absence of a mammillated coat besides the nonspherical shape of the eggs dismissed the initial presumption of *A. lumbricoides* eggs. Furthermore, the initial apparent similarities of the eggs with capillariid were ruled out based on the morphological appearance described in the literature [12]. Possible *Physaloptera* eggs have been identified in prehistoric coprolites from South America. In Argentina, these eggs were identified from canid and human coprolites [10, 11]. In Brazil, such eggs were found in feline paleofeces dating back to 9000 years [25]. Cleeland et al. by studying DNA extracted from a 1400-year-old desiccated fecal sample from La Cueva de Los Muertos Chiquitos, archeological site in Mexico, identified *Physaloptera* DNA by amplifying and sequencing an 18S ribosomal RNA gene specific to *Ascaris* [6]. In our study, 320 burial soil samples were examined, and only one sample was found to be parasitized; a much lower number than expected initially. Some biotic and abiotic factors are involved in interpreting false-negative results in paleoparasitology [20] and, particularly, the destructive role of nematophagous fungi on helminth ova [18]. In a recent paper, cultures of the soil samples obtained from the residential area of Shahr-e Sukhteh at a depth of 1.5–2 m revealed sporogenic microbes such as *Bacillus subtilis* and saprophyte fungi such as *Aspergillus flavus* and *Cladosporium sphaerospermum*, known to have been responsible for food spoilage at that time [19]. First and foremost among the limitations of the present study is that we had access to only a few eggs. Consequently, future studies in Shahr-e Sukhteh should aim to detect the possible existence of nematophagous fungi and their destructive effects leading to the disappearance of the helminth eggs over time.

The discovery of parasitic infections that are common today in archeological sites is frequent worldwide, whereas the scenario of finding rare parasites is infrequent. Nevertheless, the justification for the present *Physaloptera* spp. eggs can be sought in the inferential process of egg identification and the parasite transmission pattern. Environmental conditions, agricultural factors, and the abundance of sheep and goats, as well as arthropods like beetles, all of which have been described for Shahr-e Sukhteh in its heyday, can evidently support our present findings [4, 7, 8]. Remarkable reports on *Physaloptera* spp. in different kinds of animals globally, [5, 14] including in Iran [9, 16], especially given the very rare human cases throughout the 20th century, imply a dramatic decline in the chances of human physalopterosis in modern versus ancient times. Human occurrence has never been observed in Iran. Although poor sanitary conditions in 3200 BC should not be ignored in infection transmission, modern human case reports, although rare, can justify the occurrence of spiruridiasis at any time.
